# A Flow Cytometric and Computational Approaches to Carbapenems Affinity to the Different Types of Carbapenemases

**DOI:** 10.3389/fmicb.2016.01259

**Published:** 2016-08-09

**Authors:** Cidália Pina-Vaz, Ana P. Silva, Isabel Faria-Ramos, Rita Teixeira-Santos, Daniel Moura, Tatiana F. Vieira, Sérgio F. Sousa, Sofia Costa-de-Oliveira, Rafael Cantón, Acácio G. Rodrigues

**Affiliations:** ^1^Department of Microbiology, Faculty of Medicine, University of Porto, PortoPortugal; ^2^CINTESIS – Center for Research in Health Technologies and Information Systems, PortoPortugal; ^3^Department of Microbiology, São João Hospitalar Center, PortoPortugal; ^4^Department of Pharmacology and Therapeutic, Faculty of Medicine, University of Porto, PortoPortugal; ^5^UCIBIO, REQUIMTE, Department of Chemistry and Biochemistry, Faculty of Sciences, University of Porto, PortoPortugal; ^6^Servicio de Microbiología, Instituto Ramón y Cajal de Investigación Sanitaria, Hospital Universitario Ramón y Cajal, MadridSpain

**Keywords:** carbapenems, *Enterobacteriaceae*, docking, carbapenemases, MDR microorganisms

## Abstract

The synergy of carbapenem combinations regarding *Enterobacteriaceae* producing different types of carbapenemases was study through different approaches: flow cytometry and computational analysis. Ten well characterized *Enterobacteriaceae* (KPC, verona integron-encoded metallo-β-lactamases –VIM and OXA-48-like enzymes) were selected for the study. The cells were incubated with a combination of ertapenem with imipenem, meropenem, or doripenem and killing kinetic curves performed with and without reinforcements of the drugs. A cephalosporin was also used in combination with ertapenem. A flow cytometric assay with DiBAC4-(3), a membrane potential dye, was developed in order to evaluate the cellular lesion after 2 h incubation. A chemical computational study was performed to understand the affinity of the different drugs to the different types of enzymes. Flow cytometric analysis and time-kill assays showed a synergic effect against KPC and OXA-48 producing-bacteria with all combinations; only ertapenem with imipenem was synergic against VIM producing-bacteria. A bactericidal effect was observed in OXA-48-like enzymes. Ceftazidime plus ertapenem was synergic against ESBL-negative KPC producing-bacteria. Ertapenem had the highest affinity for those enzymes according to chemical computational study. The synergic effect between ertapenem and others carbapenems against different carbapenemase-producing bacteria, representing a therapeutic choice, was described for the first time. Easier and faster laboratorial methods for carbapenemase characterization are urgently needed. The design of an ertapenem derivative with similar affinity to carbapenemases but exhibiting more stable bonds was demonstrated as highly desirable.

## Introduction

Worldwide carbapenem resistance is growing leading to increased morbidity and mortality caused by multidrug resistant pathogens (MDR) and costs due to longer hospital stay (Patel and Bonomo, 2011; Nordmann et al., 2012; Tzouvelekis et al., 2012). Carbapenemases are enzymes that destroy almost all β-lactams. Infections caused by these bacteria are difficult to treat as only few antibiotics (including colistin, tigecycline, and aminoglycosides) may remain effective, and resistance to these compounds has also recently emerged in *Enterobacteriaceae*. Treatment with a single effective antibiotic is associated with an unacceptably high mortality rate, and combination regimens should be the rule to obtain a clear survival benefit (Tzouvelekis et al., 2014). Minimal inhibitory concentrations (MIC) >0.125 mg/L for either meropenem or ertapenem and >1 mg/mL for imipenem, require screening of carbapenemase production (EUCAST, 2013). Anderson et al. (2007) found that ertapenem susceptibility is the most sensitive indicator of KPC activity. We speculate that the ertapenem is the less effective among carbapenems because the affinity of ertapenem for carbapenemases is also the highest. Therefore, we tested the hypothesis that ertapenem could be used to maintain carbapenemases occupied allowing the other carbapenems to remain intact. Similar strategies were used regarding the association between clavulanic acid and β-lactamics drugs as it was described as subtract of β-lactamases (Neu and Fu, 1978). Pioneer studies performed using animal models of infections or experimental treatments of patients have suggested the use of carbapenem combinations for treating infections, however, they are limited (Wiskirchen et al., 2013a,b, 2014a) encompasses few enzymes and deserved further explanation.

Thus, we studied the association between ertapenem and the other three carbapenems: imipenem, meropenem, and doripenem, against different types of carbapenemases producing by *Enterobacteriaceae*, belonging to Amber classe A, B, and D. Those drugs associations were studied by the time-killing kinetic curves as well as a new flow cytometry cell analysis. We have recently developed a protocol for identification of carbapenemases in 1 hour based on flow cytometry analysis (Silva et al., 2016) with great accuracy. Additionally, a computational protein–ligand docking analysis allowed us to understand the molecular affinity of the different drugs regarding different enzymes.

## Materials and Methods

### *Enterobacteriaceae* Strains

Ten well characterized *Enterobacteriaceae* clinical strains were selected; 4 KPC (two *Klebsiella pneumoniae* KPC-2, one *Escherichia coli* KPC-3 and one *K. pneumoniae* KPC-3), four verona integron-encoded beta-metalo-lactamases (one *Enterobacter cloacae* VIM-1, one *K. pneumoniae* VIM-3, one *E. aerogenes* VIM-4, and one *K. penumoniae* VIM-4) and two OXA-like strains (*K. pneumoniae* OXA-48 and *K. pneumoniae* OXA-181).

### Chemicals

Ertapenem (Merck & Co.), doripenem (Ortho-McNeil-Janssen Pharmaceuticals), meropenem (Fresenius Kabi Pharma Portugal), imipenem (LKT Laboratories), and ceftazidime (Sigma) were reconstituted with normal saline according to the manufacturer’s instructions immediately before use; solutions were kept refrigerated, protected from light, and discarded after 8 h.

### MIC Testing

Minimal inhibitory concentrations testing regarding the four carbapenems and ceftazidime was determined according to Clinical Laboratory Standard Institute microdilution reference method (CLSI, 2014) using cation-adjusted Mueller-Hinton broth. *Pseudomonas aeruginosa* ATCC 27853 was used as quality control.

### ESBL Detection

Extended-spectrum β-lactamases (ESBL) were detected by disk diffusion agar test (both ceftazidime and cefotaxime without and with clavulanic acid).

### Time-Kill Assays

Time-kill assays were performed to compare the effect produced by meropenem, imipenem, or doripenem either alone or with ertapenem against a bacterial suspension. Approximately 1 × 10^6^ cells/mL was incubated with ertapenem (0.5 and 2 mg/L) in the absence or in the presence of 8 mg/L of meropenem, doripenem, or imipenem. Carbapenem concentrations represent usual levels in human simulated regime of continuous infusion used in critically ill patients (Abdul-Aziz et al., 2012). Ceftazidime at 100 mg/L was also incubated with ertapenem under the same conditions (Georges et al., 2012). Non-treated bacterial cells were used as controls. After 2, 4, 6, 8, and 24 h of incubation at 37°C, optical density was measured and the number of colony forming units (CFUs) quantified. These procedures were repeated after drug reinforcements each 2 h. Synergy was defined as *a* ≥ 2 log_10_ decrease in CFUs/mL between the combination and ertapenem alone; bactericidal effect was defined as *a* ≥ 3-log_10_ decrease in CFUs/mL comparing the carbapenem combination with the starting inoculum.

### Flow Cytometric Assay

For flow cytometry analysis after 2 h incubation time (from time-kill assays), 1 mL of the bacteria suspensions were stained with 0.5 μg/mL DiBAC4-(3) (Sigma), a membrane potential dye and the intensity of fluorescence measured in a FACSCalibur (BD) at FL1 (530/30nm). In the presence of depolarized cells, meaning cell lesion, the intensity of fluorescence increases.

### Computational Analysis

Computational analysis through protein–ligand docking calculations (Sousa et al., 2006, 2013) was performed to evaluate the relative binding affinity of carbapenems to different carbapenemases. Initially, a thorough validation of the docking conditions for different carbapenemases was performed, using 10 known carbapenemase complexes taken from the PDB. Docking conditions adjusted at this stage included the box size and position, number of solutions, and the exhaustiveness parameter, until the docking results were able to reproduce the known X-ray poses with a maximum rmsd of 1 Å. The same docking conditions were later used in docking ertapenem, doripenem, meropenem, and imipenem against the structures of 13 different carbapenemase enzymes from classes A (five enzymes), B (three enzymes), and D (five enzymes) taken from the Protein Data Bank (see details and results in **Table 2**). This procedure was repeated with two different docking programs (Autodock Vina and GOLD) (Jones et al., 1997; Trott and Olson, 2010) and with five independent scoring functions (VINA, ChemPLP, ASP, GoldScore, and ChemScore) to evaluate the corresponding binding affinities. Average values of each scoring function for each carbapenemase-carbapenem complex were calculated and the statistical significance of the different predictions for each carbapenem was determined through a two-tailed *t*-test.

## Results and Discussion

**FIGURE 1:**
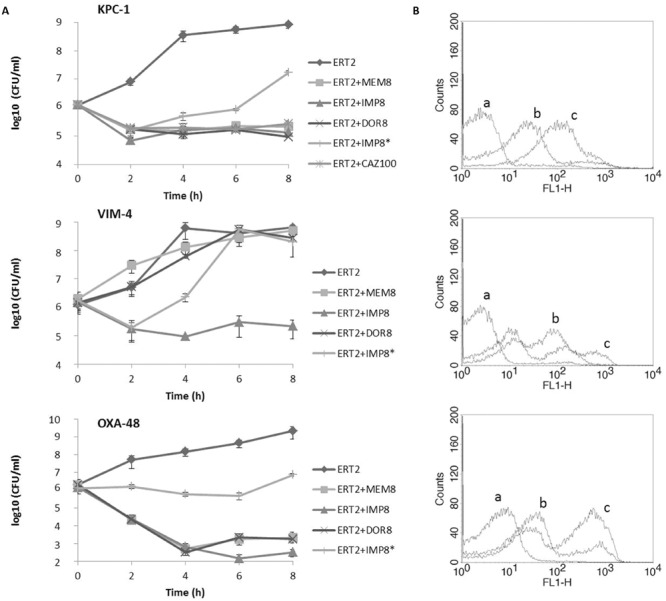
**(A)** Time-kill curves of various antibiotic combinations against a strain of *Klebsiella pneumoniae* KPC-2, a strain of *Enterobacter cloacae* VIM-4, and a *K. pneumoniae* OXA-48. Each line represents ertapenem (ERT) either alone or associated with meropenem (MEM), imipinem (IMP), or doripenem (DOR) reinforced with the respective drugs each 2 h. Ertapenem plus imipenem (*) is shown as an example of the treatment without reinforcement. The association of ERT 2 mg/L with ceftazidime (CAZ) 100 mg/L against KPC-2 producing bacteria is also represented. **(B)** Overlay of histograms obtained by flow cytometry. For each strain, cells stained with DiBAC4(3) are represented after 2 h treatment with ERT 2 mg/L (a), ERT 0.5 mg/L plus IMP 8 mg/L (b), and ERT 2 mg/L plus IMP 8 mg/L (c).

Phenotypic profiles of the strains used in the study are described in **Table 1**. None of the carbapenems alone produced a significant reduction of CFUs number or membrane depolarization, compared to controls. **Figure 1** shows time-kill curves and flow cytometry results of typical examples of each type of carbapenemase-producing bacteria. Whenever the cells were incubated with ertapenem associated with other carbapenem, a synergistic effect was evident soon after 2 h with meropenem, doripenem, or imipenem on all KPC- and OXA-producing strains and a bactericidal effect at 4 h (**Figure 1A**). In VIM-producing strains this effect was observed only with imipenem (**Figure 1A**). A sustained reduction of CFUs counts was observed only if treatment was reinforced every 2 h (**Figure 1A**); effects at 24 h were similar to 8 h effect. The association between ertapenem and other carbapenem was bactericidal only on OXA-producing strains (**Figure 1A**). A synergistic effect between ertapenem and ceftazidime was also achieved in ESBL-negative KPC strains (**Figure 1A**). No synergism was observed in ESBL co-producers using that combination. The synergistic effect was ertapenem dose-dependent (**Figure 1B**). Flow cytometric analysis corroborated CFUs results, showing cell depolarization after treatment with ertapenem associated with each different carbapenem, for KPC and OXA enzymes, but only with ertapenem/imipenem for VIM-4 enzyme (**Figure 1B**). Results obtained with VIM-1 and VIM-3 were similar. Protein–ligand docking calculations with the different scoring functions showed that ertapenem had the highest affinity, whereas imipenem had the lowest affinity toward carbapenemases (**Table 2**). This tendency was observed with all the five independent scoring functions used in this study and for all the 13 different carbapenamase enzymes considered. For VINA, ChemScore, and GoldScore the significance level of this difference was over 90%.

**Table 1:** Acquired-carbapenemase-producing isolates tested.

		MIC (mg/L)					
Carbapenemase (no. of isolates)	Species	ERT	IMP	MEM	DOR	CAZ	ESBL
KPC (4)	*K. pneumoniae*	128–256	16–32	32–128	32–64	512	-
VIM (4)	*E. cloacae*	64–256	32–64	64–128	64–128	>512	+
OXA (2)	*K. pneumoniae*	4–8	4–8	4–8	4	16	+

*ERT, ertapenem; IMP, imipenem; MEM, meropenem; DOR, doripenem; CAZ, ceftazidime.*

**Table 2:** Results of the application of five independent scoring functions in the evaluation of the binding ability of the four carbapenems tested, against 13 X-ray structures of different carbapenemase enzymes from Classes A, B, and D.

		Vina^a^	ChemPLP^b^	ASP^b^	GoldScore^b^	ChemScore^b^
**Class A** (3BFC, 3RXW, 1BUL, 3NI9, 4MXH)	Ertapenem	**-7.9 ± 0.4**	**71.0 ± 8.7**	**30.9 ± 5.1**	60.6 ± 5.8	**34.7 ± 3.5**
	Doripenem	**-**7.7 ± 0.2	64.4 ± 10.8	27.6 ± 4.7	**62.2 ± 5.3**	32.5 ± 1.1
	Meropenem	**-**7.8 ± 0.4	64.3 ± 7.2	26.6 ± 3.2	56.9 ± 4.0	30.0 ± 1.6
	Imipenem	**-**6.7 ± 0.2	62.9 ± 4.7	21.2 ± 3.3	51.9 ± 9.1	27.6 ± 1.8
**Class B** (3WXC, 2YZ3, 4EYB)	Ertapenem	**-7.7 ± 0.8**	103.3 ± 12.7	**46.7 ± 2.6**	**80.9 ± 6.9**	46.5 ± 7.6
	Doripenem	**-**7.5 ± 0.6	**109.0 ± 5.7**	43.5 ± 1.9	75.9 ± 3.0	**46.7 ± 5.0**
	Meropenem	**-**7.5 ± 0.5	105.0 ± 6.8	43.0 ± 1.7	70.5 ± 5.4	44.3 ± 4.4
	Imipenem	**-**6.4 ± 0.5	98.6 ± 8.7	34.5 ± 2.4	76.8 ± 3.7	42.8 ± 3.1
**Class D** (3ISG, 4MLL, 4JF4, 3LCE, 3ZNT)	Ertapenem	**-8.8 ± 0.9**	**76.0 ± 4.6**	**36.3 ± 4.5**	**78.2 ± 6.3**	**33.3 ± 3.3**
	Doripenem	**-**7.6 ± 0.3	70.7 ± 5.0	33.4 ± 2.4	72.1 ± 4.1	31.0 ± 3.2
	Meropenem	**-**7.5 ± 0.5	68.5 ± 3.7	32.4 ± 3.3	70.0 ± 4.1	29.4 ± 3.8
	Imipenem	**-**6.5 ± 0.7	65.2 ± 6.0	27.4 ± 3.4	65.8 ± 5.7	26.7 ± 3.2
*t*-test (ertapenem vs imipenem)^c^		**100.0%**	**55.1%**	**99.7%**	**92.7%**	**77.4%**

*PDB codes given in parenthesis. Highest affinity ligand for each scoring function indicated in bold. ^a^The Vina Scoring function reflects the predicted binding affinity in kcal/mol (Trott and Olson, 2010). ^b^The ChemPLP, ASP (the Astex Statistical Potential), GoldScore, and ChemScore scoring functions are dimensionless, however, in each case, the scale of the score gives a guide as to how good the pose is. The higher the score, the better the docking result is likely to be (Jones et al., 1997). ^c^Statistical significance of the average scoring values for ertapenem and imipenem being statistically different calculated from a two-tailed *t*-test.*

There were no marked differences between the three carbapenemase families tested. The computationally predicted binding affinities between ertapenem and enzymes suggest weak and reversible binding. A combination of ertapenem with doripenem shows promising results regarding a KPC *K. pneumoniae* strain both *in vitro* and *in vivo* (Bulik and Nicolau, 2011). Wiskirchen et al. (2013a,b, 2014b) also showed in a murine infection model a large variation of efficacy of carbapenem monotherapy against bacteria producing KPC, NDM-1, and OXA-48 enzymes. In immunocompetent mice doripenem (2 g q8h) with ertapenem (1 g qd) increased the efficacy over doripenem alone for the isolate with a doripenem MIC of 8 μg/mL (Bulik and Nicolau, 2011). In our study, imipenem with ertapenem showed synergy against all kinds of carbapenemase-producing bacteria. Meropenem or doripenem associated to ertapenem were not active against VIM. VIM was the only MBL studied. Therefore extrapolation to other subtypes (for instance, IMP) requires further studies despite structural similitudes. The highest synergistic effect was observed in OXA producing-strains in agreement with Wiskirchen et al. (2014a) that describe a higher efficacy of dual carbapenem regimes when MICs are low. A different approach was taking by Poirel et al. (2016) and their data strongly support the hypothesis that dual carbapenem combinations might be effective against serine-b-lactamase producers (KPC, OXA-48). And the imipenem-containing combinations appeared to be the most efficient. Rationale of using ertapenem plus other carbapenems in carbapenemase producing *Enterobacteriaceae* is similar to the use of clavulanate with amoxicillin on TEM-1 producers. However, in our case, clavulanate has no inhibitory effect on carbapenemases. The only one in which this inhibitory effect is slightly present is with KPC enzymes but this inhibition is far from clinical use. In the future, this assay can be eventually compared with ceftazidime/avibactam combination (only with KPC or OXA-48-like enzymes) (Chahine et al., 2015). Flow cytometry is an excellent tool, still unexplored in Microbiology, allowing to study antimicrobial activity (Pina-Vaz et al., 2005; Pina-Vaz and Rodrigues, 2010) drug associations (Teixeira-Santos et al., 2012), and mechanisms of resistance (Faria-Ramos et al., 2013; Silva et al., 2016). Flow cytometric results showed that even at a low concentration and a short incubation time, a synergistic effect could be easily observed and quantified.

## Conclusion

We concluded that all carbapenemases easily hydrolyze ertapenem allowing the other carbapenems to reach their bacterial targets. These results turn important the characterization of the carbapenemase present on the strain which is not performed by all the clinical laboratories; the available methods are cumbersome, expensive and gave late results. Easier and faster laboratorial methods are need.

## Author Contributions

All authors listed, have made substantial, direct and intellectual contribution to the work, and approved it for publication.

## Conflict of Interest Statement

The authors declare that the research was conducted in the absence of any commercial or financial relationships that could be construed as a potential conflict of interest. The reviewer WP and handling Editor declared their shared affiliation, and the handling Editor states that the process nevertheless met the standards of a fair and objective review.
